# Left atrial structure and function in heart failure with reduced (HFrEF) versus preserved ejection fraction (HFpEF): systematic review and meta-analysis

**DOI:** 10.1007/s10741-021-10204-8

**Published:** 2022-01-26

**Authors:** Xuanyi Jin, Jan F. Nauta, Chung-Lieh Hung, Wouter Ouwerkerk, Tiew-Hwa Katherine Teng, Adriaan A. Voors, Carolyn SP. Lam, Joost P. van Melle

**Affiliations:** 1 National Heart Centre Singapore, Singapore, Singapore; 2 Department of Cardiology, University of Groningen, University Medical Centre Groningen, Hanzeplein 1, 9700 RB Groningen, the Netherlands; 3 Duke-NUS Medical School, Singapore, Singapore; 4 Division of Cardiology, Department of Internal Medicine, Mackay Memorial Hospital, Taipei, Taiwan; 5 Department of Dermatology, Netherlands Institute for Pigment Disorder/Amsterdam Infection & Immunity Institute; Cancer Center Amsterdam, Amsterdam, the Netherlands; 6 Institute of Biomedical Sciences, Mackay Medical College, New Taipei City, Taiwan

**Keywords:** LA structure, Function, HFrEF, HFpEF

## Abstract

Left atrial (LA) structure and function in heart failure with reduced (HFrEF) versus preserved ejection fraction (HFpEF) is only established in small studies. Therefore, we conducted a systematic review of LA structure and function in order to find differences between patients with HFrEF and HFpEF. English literature on LA structure and function using echocardiography was reviewed to calculate pooled prevalence and weighted mean differences (WMD). A total of 61 studies, comprising 8806 patients with HFrEF and 9928 patients with HFpEF, were included. The pooled prevalence of atrial fibrillation (AF) was 34.4% versus 42.8% in the acute inpatient setting, and 20.1% versus 33.1% in the chronic outpatient setting when comparing between HFrEF and HFpEF. LA volume index (LAVi), LA reservoir global longitudinal strain (LAGLS_R_), and E/e’ was 59.7 versus 52.7 ml/m^2^, 9.0% versus 18.9%, and 18.5 versus 14.0 in the acute inpatient setting, and 48.3 versus 38.2 ml/m^2^, 12.8% versus 23.4%, and 16.9 versus 13.5 in the chronic outpatient setting when comparing HFrEF versus HFpEF, respectively. The relationship between LAVi and LAGLS_R_ was significant in HFpEF, but not in HFrEF. Also, in those studies that directly compared patients with HFrEF versus HFpEF, those with HFrEF had worse LAGLS_R_ [WMD = 16.3% (22.05,8.61); *p* < 0.001], and higher E/e’ [WMD = −0.40 (−0.56, −0.24); *p* < 0.05], while LAVi was comparable. When focusing on acute hospitalized patients, E/e’ was comparable between patients with HFrEF and HFpEF. Despite the higher burden of AF in HFpEF, patients with HFrEF had worse LA global function. Left atrial myopathy is not specifically related to HFpEF.

**Supplementary Information:**

The online version contains supplementary material available at 10.1007/s10741-021-10204-8.

## Introduction

The left atrium can be considered a transporting chamber that optimizes left ventricular (LV) filling [1]. Left atrial (LA) hypertension with subsequent pulmonary venous congestion is the hallmark of HF regardless of LV ejection fraction (LVEF) [2, 3]. More recently, the significant pathophysiological role of LA dysfunction in HF has gained increasing attention, particularly in HF with preserved EF (HFpEF) [3–5]. Over the past decades, the incidence of HFpEF has risen relative to HF with reduced ejection fraction (HFrEF), accounting now for approximately 50% of cases of HF [6, 7]. Studies have shown that atrial fibrillation (AF), diabetes, and obesity are risk factors for the development of HFpEF, whereas coronary artery disease (CAD) and myocardial infarction are more predisposed to the development of HFrEF [6, 7]. The close link between AF and HFpEF might be explained by intrinsic LA myopathy underlying both HFpEF and AF [8].

However, information regarding differences in LA structure and function between HFrEF and HFpEF, particularly LA functional information assessed by strain analysis, is scarce and not fully understood. Thus, we aimed to conduct a systematic review of LA structure and function assessed by echocardiography in patients with HFrEF versus HFpEF.

## Methods

The systemic review and meta-analysis were conducted according to the Preferred Reporting items for Systemic Reviews and Meta-Analysis (PRISMA) statement [9]. The review protocol had been registered with PROSPERO (http://www.crd.york.ac.uk/PROSPERO).

### Literature search strategy

We performed a systematic search in the MEDLINE and EMBASE database from inception through February 2021. Our search was restricted to studies in the English language. Additional studies were selected by reviewing and searching references of identified articles, which were not identified by the initial search. Search terms are mainly composed of the patient domain, including “heart failure,” “heart failure with preserved ejection fraction” and “heart failure with reduced ejection fraction,” and outcome domain as LA structure and function related terms, respectively. The detailed search strategy was described in the online supplementary Table S1.

### Study selection

Studies were eligible if they were performed in a clearly defined group of patients with HFrEF or HFpEF or both. The study population had to have a clinical diagnosis of HF, based on signs and symptoms such as dyspnea, fatigue at rest or during exercise, or a previous HF hospitalization. At least one measure of LA structure and function assessed by echocardiography had to be reported. For HFrEF versus HFpEF categorization, the cutoff value of LVEF assessed by echocardiography had to be 45% or 50%. Elevated natriuretic peptides were recognized, but not mandatory for study inclusion. Two authors (XY.J, K.TH.T) independently screened the titles and abstracts of retrieved citations to identify potentially relevant studies. If abstracts were ambiguous, studies were reviewed at the full-text level. Citations were included when consensus between two authors was achieved.

### Data extraction

For each included study, the following data of study participants were extracted: (1) baseline characteristics [i.e., publication year, the total number of study participants, the clinical setting of HF (i.e., inpatient vs outpatient setting), age, sex, body mass index (BMI), hypertension, ischemic heart disease (IHD), atrial fibrillation (AF), diabetes, and presence of more than moderate functional mitral regurgitation (MR)], (2) echocardiographic characteristics [i.e., LVEF, LV global longitudinal strain (GLS), the ratio of mitral valve peak velocity of early and late LV filling (E/A), mitral annulus e’ velocity (e’), E/e’ ratio, LA (reservoir, booster, conduit) GLS, software used for post-offline analysis]. When longitudinal studies reported cardiovascular outcomes (mortality and hospitalization), unadjusted and adjusted hazard ratio (HR) for the association between the LA-related parameter with outcomes were obtained. Follow-up time in months, outcome measure, and variables for which was adjusted were also obtained.

### Quality assessment

To perform a quality assessment of included studies, the Newcastle–Ottawa scale adapted for observational studies [10] was used scoring each study on several items (i.e., selection process, comparability, and assessment of the outcome/exposure criterion). Moreover, the quality of the clinical trials was evaluated using the revised Cochrane risk-of-bias tool (RoB 2.0) [11], covering five domains (randomization, intervention, missing data, outcome measure, and reported results) of included studies.

### Statistical analysis

Continuous variables were reported as mean ± standard deviation (SD), and categorical variables as percentage. When only medians and interquartile ranges were reported in the study, we translated those into means and SDs by an established formula based on previous recommendations [12].

The summary and pooled values of corresponding LA parameters were calculated by the weighted average based on the number of patients among included studies and depicted in forest plots for HFrEF and HFpEF, respectively. The prevalence of comorbidities for included studies was pooled by the weighted average according to the number of patients for HFrEF and HFpEF, respectively. Data on LA related echocardiographic parameters in both patients with HFrEF and HFpEF were pooled to derive weighted mean differences (WMDs) and 95% confidence intervals (CI). Linear regression and the mixed-effects meta-regression model were applied to investigate the relationship of LAGLS_R_ with LAVi and LVGLS in patients with HFrEF and HFpEF, respectively. Random effects model with inverse variance weighting was performed using the Cochrane I^2^ statistic to account for heterogeneity across the studies. All statistical analyses were performed using RStudio version 1.1456.

## Results

### Study characteristics and quality assessment

**Fig. 1:**
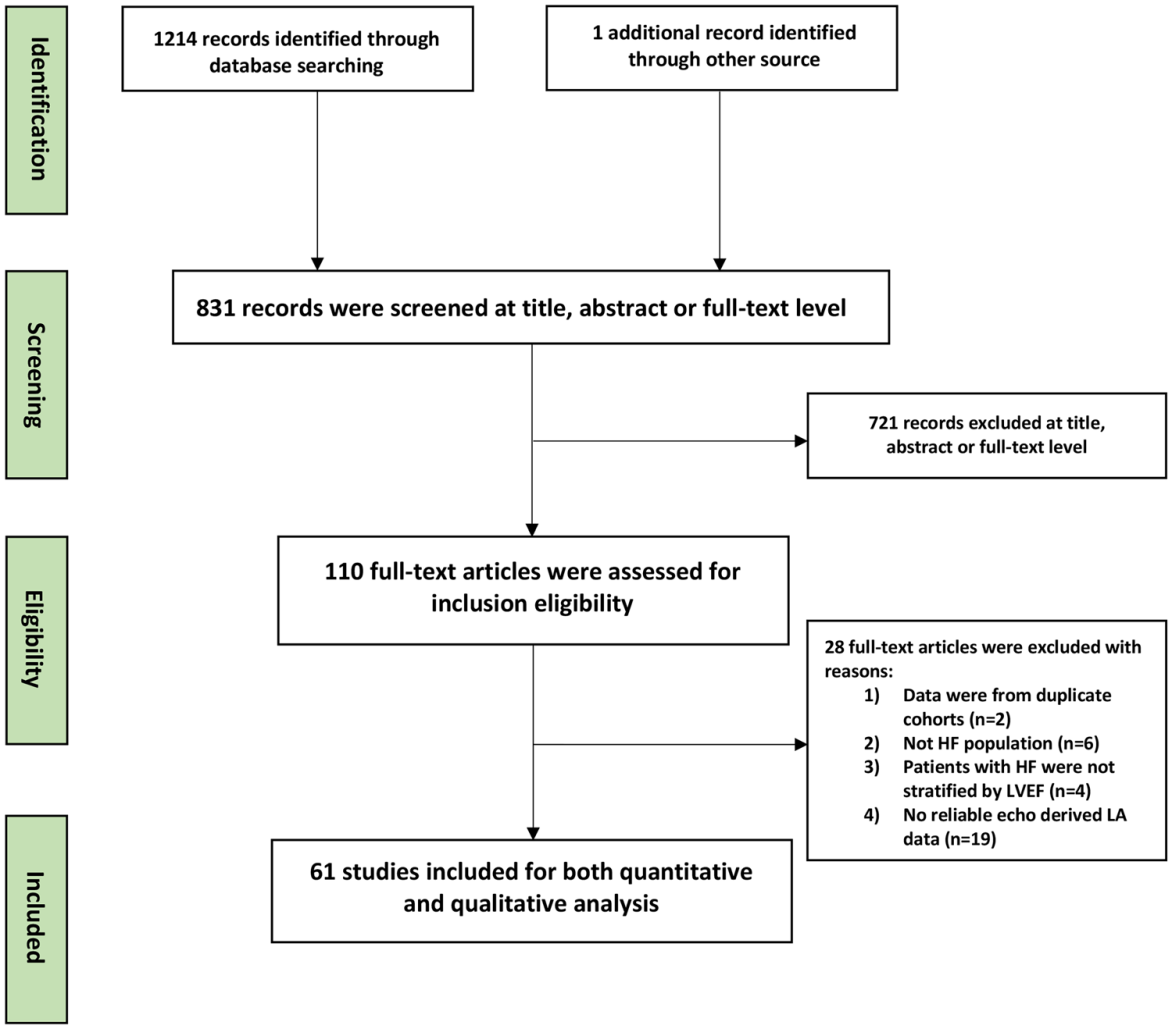
PRISMA flowchart of process for literature search and study selection. HF, heart failure; LA, left atrial, LVEF, left ventricular ejection fraction

The search strategy and study selection are summarized in the PRISMA flowchart [9] (Fig. 1). Of 1114 studies identified, a total of 61 studies were selected for the final quantitative and qualitative analysis. The quality assessment of included studies is shown in the supplementary material online (Tables S2 and S3). Reasons for exclusions were described in the supplementary Table S4. Among 61 studies, 27 studies (including 8806 patients with HFrEF and 38 studies including 9928 patients with HFpEF) reported LA structural and functional parameters by echocardiography. Nine out of 61 studies included both patients with HFrEF (*n* = 1877) and HFpEF (*n* = 3085). Nine out of 61 studies included patients with HF from an acute inpatient setting (HFrEF, *n* = 2749; HFpEF, *n* = 3319), whereas fifty-two studies included patients with HF from a chronic stable outpatient setting (HFrEF, *n* = 6057; HFpEF, *n* = 6714). The pooled clinical and echocardiographic characteristics in patients with HFrEF versus HFpEF in the acute inpatient versus chronic outpatient setting were described separately in Table 1. Moreover, the details of clinical and echocardiographic characteristics of included studies are described in Tables 2 and 3.

**Table 1:** The pooled clinical and echocardiographic characteristics in patients with HFrEF versus HFpEF

	Acute inpatient setting		Chronic outpatient setting	
	HFrEF (*n* = 2749)	HFpEF (*n* = 3319)	HFrEF (*n* = 6057)	HFpEF (*n* = 6714)
Age (years)	69.0	73.0	60.8	67.3
Sex (female, %)	37.8%	57.8%	28.7%	58.9%
Diabetes (%)	36.6%	37.1%	28.2%	33.2%
AF (%)	34.4%	42.8%	20.1%	33.1%
IHD (%)	39.8%	30.7%	49.8%	33.3%
BMI (kg/m^2^)	25.2	25.6	27.5	29.8
Presence of moderate to severe mitral regurgitation (%)	-	-	27.2%	12.0%
LVEF (%)	25.6	60.1	27.9	61.8
LVGLS (%)	−12.5	−15.1	−8.4	−16.5
MV e’(cm/s)	4.7	6.6	6.5	7.5
E/e’	18.5	14.0	16.9	13.5
LAVi (ml/m^2^)	59.7	52.7	48.3	38.2
LAGLS_R_ (%)	9.0	18.9	12.8	23.4
LAGLS_B_ (%)	-	-	7.7	13.9
LAGLS_C_ (%)	-	-	-	15.8

*HFrEF* heart failure with reduced ejection fraction, *HFpEF* heart failure with preserved ejection fraction, *AF* atrial fibrillation, *IHD* ischemic heart disease, *BMI* body mass index, *LVEF* left ventricle ejection fraction, *LVGLS* left ventricle global longitudinal strain, *MV e’* mitral annular early diastolic velocity by tissue doppler, *E/e’* the ratio between early mitral inflow velocity and mitral annular early diastolic velocity, *LAVi* left atrial volume index, *LAGLS*_*R*_ left atrial global longitudinal strain at reservoir phase, *LAGLS*_*B*_ left atrial global longitudinal strain at booster phase, *LAGLS*_*C*_ left atrial global longitudinal strain at conduit phase

**Table 2:** Clinical characteristics of included studies

Author/year	Study design	Study setting	Heart failure phenotype examined	LVEF cutoff	Number of patients (*n*)	Age (years)	Female sex (%)	Atrial fibrillation (%)	Diabetes mellitus (%)	Hypertension (%)	Ischemic heart disease (%)	BMI (kg/m^2^)	Moderate to severe mitral regurgitation	LA structure and functional parameters measured
Hoshida et al. [27]	Prospective multi-center observational study	CHF, inpatient setting	HFpEF	≥ 50%	105	78.5 ± 10.2	53.3%		41%	88%		24.3 ± 5.0		LAVi, E/e'
Harada et al. [28]	Prospective single-center cohort study	AHF, compensatory inpatient setting	HFpEF	≥ 45%	92	73.0 ± 12.8	59%	47%	27%	72%	34%	22.3 ± 3.6		LAVi, E/e', e'
Hwang et al. [29]	Prospective multi-center observational study	AHF, multi-center, inpatient setting	HFpEF	≥ 50%	1105	76.0 ± 9.6	60.6%	32.9%	32.4%	64.3%	29.3%	23.9 ± 3.7		LAVi, E/e' LA reservoir strain
Shah et al. [30]	Retrospective cohort study	CHF, inpatient setting	HFrEF	≤ 40%	67	49.5 ± 11.4	34.3%	9%		64.2%	35.8%	31.8 ± 7.0		LAVi, E/e'
			HFrEF	≤ 40%	69	57.5 ± 15.3	24.6%	8.7%		60.9%	36.2%	31.1 ± 7.3		LAVi, E/e'
Tanaka et al. [31]	Retrospective cohort study	CHF, outpatient setting	HFrEF	≤ 45%	205	59 ± 17	31%	14%	27%	19%			32%	LAVi, E/e'
Castrichini et al. [32]	Prospective single-center cohort study	CHF, outpatient setting	HFrEF	< 40%	77	65 ± 11	12.1%	37.7%	45.5%	54.5%	40.3%		32%	LAVi, E/e', LA reservoir strain
Valentim et al. [33]	Prospective single-center cohort study	CHF, outpatient setting	HFrEF	< 40%	42	58.6 ± 11.1	17.1%	40%	31.4%		42.9%	28.1 ± 3.8		LAVi, E/e'
Kurzawski et al. [34]	Retrospective single-center cohort	CHF, inpatient and outpatient settings	HFrEF	< 25%	63	61.9 ± 10.9	4.8%		33.3%	54%	52.4%	26.2 ± 4.5		LAVi, E/e', LA reservoir strain
Park et al. [16]	Retrospective cohort study	AHF, multi-center, inpatient setting	HFpEF	≥ 50%	1191	73.4 ± 13.3	60.3%	35%	31%	62%	27.4%	23.8 ± 4.1		LAVi, E/e', LA reservoir strain, e'
			HFrEF	< 40%	2036	68.4 ± 14.1	38.3%	24.9%	36.3%	54.2%	34.4%	23.1 ± 4.3		
Deferm et al. [35]	Prospective single-center cohort study	ADHF (Acute decompensated HF), inpatient setting	HFrEF	≤ 40%	31	64 ± 15	22.6%	32.3%	29%	48.4%	48.4%	28.1 ± 6.0	51.6%	LAVi, E/e' LA reservoir strain
Shah et al. [36]	Randomized, multi-center double-blind placebo controlled trial	CHF, multi-centers (752 sites in 43 countries), inpatients and outpatient settings	HFpEF (PARAGON-HF trial-ECHO study	≥ 45%	1097	74 ± 8	53%	35%	40%	94%	30%	29.9 ± 4.9	12%	LAVi, E/e', E/A, e'
Reddy et al. [14]	Prospective single-center cohort study	CHF, single-center, outpatient setting	HFpEF	≥ 50%	238	68 ± 10	62%	17%	29%	90%	32%	32.9 ± 7.1		LAVi, LA Reservoir, conduit and contractile strain
Modin et al. [18]	Retrospective single-center cohort study	CHF, outpatient setting	HFrEF	≤ 45%	818	66.4 ± 11.4	26.6%	15.3%	11.4%	41.2%	55.9%	26.4 ± 4.8	9%	LAVi, E/e', E/A, e'
Shintani et al. [37]	Retrospective single-center cross-sectional study	AHF, inpatient setting	HFpEF	≥ 50%	127	80.6 ± 8.1	50%	52%	41%	67%		23.2 ± 3.7		LAVi
			HFrEF	< 40%	617	75.0 ± 11.1	33%	48%	37%	71%		22.5 ± 3.7		
Wu et al. [38]	Prospective single-center cohort study	CHF, inpatient setting	HFpEF	≥ 50%	163	61.1 ± 15.3	61%		30.1%	60.1%	3.5%	25.9 ± 4.2		LAVi, E/e', E/A, e'
			HFrEF	< 40%	34	54.5 ± 15.4	18%		17.6%	44.1%	44.1%	25.2 ± 4.7		
Telles et al. [39]	Prospective single-center cohort study	CHF, inpatient setting	HFpEF	≥ 50%	49	69.4 ± 8.0	71.4%	26.5%	14%	67%	14%	30.2 ± 5.0		LAVi, E/e', LA reservoir strain, conduit, e', E/A
Sobirin et al. [40]	A single-center, unblind, randomized, controlled clinical trial	CHF, outpatient setting	HFpEF	> 50%	30	62 ± 8	50%		73.3%					LAVi, E/e'
Lundberg et al. [41]	Prospective single-center cohort study	CHF, inpatient setting	HFpEF	≥ 50%	92	73.0 ± 8.8	62%	48%	19%	69%	3%	26.6 ± 5.2		LAVi, E/e', LA reservoir strain, e', E/A
			HFrEF	< 50%	72	56.3 ± 12.6	21%	46%	18%	58%	26%	27.3 ± 5.2		
Saikhan et al. [42]	Prospective single-center cohort study	CHF, outpatient setting	HFpEF	≥ 50%	110	63 ± 11	38.1%	excluded	48.1%	82.7%	60%	27.8 ± 5.4		LAVi, LA Reservoir, conduit and contractile strain
Burns [43]	Prospective single-center cohort study	CHF, outpatient setting	HFpEF with anemia	≥ 50%	224	65 ± 12	56%	26%	37%	79%	50%	32 ± 10		LAVi, E/e', e'
			HFpEF without anemia	≥ 50%	195	63 ± 13	69%	27%	28%	75%	46%	33 ± 9		
Obokata et al. [44]	Prospective single-center cohort study	CHF, inpatient and outpatient settings	HFpEF	≥ 50%	271	71 ± 9	56%	42%	33%	84%	57%	32 ± 7		LAVi, E/e', e'
Nagy et al. [45]	Subset of prospective, observational, multi-center study	CHF, inpatient setting	HFpEF	≥ 45%	86	72 ± 10	51%	60%	33%	79%	15%	30 ± 5		LAVi, E/e', LA reservoir strain, e, E/A ratio'
Carluccio et al. [19]	Prospective single-center cohort study	CHF, outpatient setting	HFrEF	≤ 40%	405	65.2 ± 12.3	24%		26%		38%	26.6 ± 4.1		LAVi, E/e', LA reservoir strain, e, E/A ratio'
Malagoli et al. [46]	Prospective single-center cohort	CHF, outpatient setting	HFrEF	< 40%	286	67 ± 11	19%				64%			LAVi, LA Reservoir strain
Eroglu et al. [47]	Retrospective cohort	CHF, outpatient setting	HFrEF	< 50%	59	57 ± 13	23%				84%			LAVi, E/e', E/A, e'
Almeida et al. [48]	Retrospective case–control study	AHF, inpatient setting	HFpEF	≥ 50%	65		55%		47.7%	80%	33.8%			LAVi, E/e'
			HFrEF	< 40%	65				43.1%	70.8%	44.6%			
Liu et al. [49]	Prospective single-center study	CHF, inpatient setting	HFpEF	≥ 50%	55	61 ± 13	54.5%		43%	93%	33%			LAVi, E/e', LA reservoir strain, e', E/A
Shah et al. [50]	Prospective multinational multi-center observational study	CHF, outpatient setting	HFpEF	≥ 40%	51	72.4 ± 9.0	63%	35%	25%	92%	16%	32.5 ± 10.7		LAVi, E/e', LA reservoir strain
		CHF, outpatient setting	HFpEF	≥ 40%	151	74.7 ± 8.7	52%	58%	30%	81%	21%	29.0 ± 8.5		LAVi, E/e', LA reservoir strain
Xu et al. [51]	Retrospective, single-center cohort	CHF, inpatient setting	HFrEF	< 40%	28	38 ± 14	18%					20.6 ± 3.2	57.1%	
		CHF, inpatient setting	HFrEF	< 40%	17	42 ± 10	41%					22.5 ± 5.2	17.6%	
Saha et al. [52]	Retrospective, single-center cohort	CHF, outpatient setting	HFrEF	< 40%	49	72 ± 13	42%		8%	12%	68%			E/e', LA reservoir strain
Abohammar et al. [53]	Prospective single-center observational study	AHF, inpatient setting	HFpEF	> 50%	114	59 ± 8	55%		64%	64%	16%	27 ± 3		LAVi, E/e'
Modin et al. [54]	Retrospective single-center cohort	CHF, outpatient setting	HFrEF	< 45%	151	70.5 ± 9.2	21.2%		9.2%		43%	26.7 ± 5.1		LAVi, E/e'
Batalli [55]	Prospective single-center cohort	CHF	HFpEF	NA	55	63.0 ± 6.8		Excluded		59%	41%	29 ± 4		E/e'
			HFrEF	NA	56	62 ± 12		Excluded		38%	45%	28.0 ± 3.6		
Sugimoto et al. [56]	Prospective single-center study	CHF, outpatient setting	HFpEF	> 50%	20	72.6 ± 10.3	60%		42%	74%	10%	28.3 ± 5.0		LAVi, E/e', LA reservoir strain, E/A
			HFrEF	< 40%	49	63.1 ± 12.9	31%		35%	63%	52%	26.7 ± 4.5		
Hage et al. [57]	Subset of prospective observational multicenter study	AHF, inpatient setting	HFpEF	> 45%	86	72.3 ± 8.9	51%	57%	31%	79%	34%	28.8 ± 5.9		LAVi, E/e'
Sargento et al. [58]	Prospective single-center observational study	CHF, outpatient setting	HFrEF	< 40%	203	67.8 ± 12.5	26.6%	26.1%	32%	88.7%	39.4%	27.2 ± 4.4		LAVi
Aung et al. [59]	Prospective two center study	CHF	HFpEF	≥ 50%	38	65.2 ± 5.7	50%		13.2%	60.5%	47.4%	28.1 ± 2.0		LAVi, E/e', LA reservoir strain, contractile, e'
Hung [60]	Prospective single-center cohort study	CHF, outpatient setting	HFpEF	≥ 50%	58	64.3 ± 12.4	53.4%		32.8%	74.1%		27.2 ± 3.7		E/e', LA reservoir strain, e', E/A
Freed et al. [61]	Prospective single-center cohort study	CHF, outpatient setting	HFpEF	≥ 50%	308	65 ± 13	64%	26%	30%	75%	50%	31.5 ± 8.6	14%	LAVi, LA Reservoir, conduit and contractile strain, E/e', E/A
Unger et al. [62]	Prospective single-center cohort	CHF, outpatient setting	HFpEF without CKD	> 50%	154	60.9 ± 12.3	62%	22%	21%	68%	46%	31.8 ± 8.7		LAVi, E/e', LA reservoir, conduit and booster strain
			HFpEF with CKD	> 50%	145	69.3 ± 12.1	66%	30%	39%	83%	52%	31.5 ± 8.4		LAVi, E/e', LA reservoir, conduit and booster strain
Georgievska-Ismail et al. [63]	Prospective single-center, cross-sectional study	CHF, outpatient setting	HFpEF	> 50%	108	63.2 ± 8.9	60.2%			98.1%	18.5%	29.9 ± 3.8		
Melenovsky et al. [17]	Retrospective single-center cohort study	CHF, outpatient setting	HFpEF	≥ 50%	101	71 ± 10	58%	42%	47%	93%	44%	34.0 ± 8.6		LAVi,e'
			HFrEF	< 50%	97	61 ± 13	20%	26%	41%	56%	46%	31.0 ± 6.9		
Gracia et al. [64]	Prospective single-center cohort study	CHF, outpatient setting	HFpEF	≥ 50%	28	60 ± 2			40%	90%				LAVi, E/e', E/A, e'
Hasselberg et al. [65]	Prospective single-center Cross-sectional study	CHF, inpatient setting	HFpEF	≥ 50%	37	58 ± 11	32.4%		14%	41%	60%	26 ± 4		LAVi, E/e', E/A, e'
Sanchis et al. [15]	Prospective single-center cohort	CHF, outpatient setting	HFpEF	≥ 50%	63	76 ± 8	71.4%	39.7%	23.8%	85.7%		30 ± 5		LAVI, E/e', LA reservoir strain
Shah et al. [66]	International, multicenter, randomized, double blind placebo-controlled trial (with an echo substudy)	CHF, multi-center (270 sites in 6 countries)	HFpEF (TOPCAT-ECHO)	≥ 45%	935	69.9 ± 9.7	49%	38%	40%	91%	60%	32.6 ± 7.5		LAVi, E/e', LA reservoir, contractile strain, E/A, e'
Santos et al. [67]	Echo substudy multicenter, international, randomized, double blind placebo-controlled trial	CHF, multi-centers (65 centers in 13 countries)	HFpEF (PARAMOUNT trial)	≥ 45%	135	70 ± 9	61%	23%	35%	92%	22%	29.6 ± 5.7		LAVi, LA Reservoir, conduit and contractile strain, E/e', E/A
			HFrEF	< 40%	32	74 ± 12	37.5%	50%	43.8%	78.1%				
Donal et al. [68]	Prospective, multi-center international observational study	AHF inpatient setting	HFpEF	≥ 45%	539	77 ± 19	56%		30%	78%	43.5%	29 ± 6		LAVi, E/e', E/A, e'
Burke et al. [69]	Prospective single-center cohort	CHF, outpatient setting	HFpEF	≥ 50%	419	65 ± 13	62%	26%	33%	77%	48%	33 ± 9	14%	LAVi, E/e', e'
Motoki et al. [70]	Prospective single-center cohort	CHF, outpatient setting	HFrEF	≤ 35%	108	57 ± 15	23%	excluded	27%	51%	45%			LAVi, E/e', LA reservoir strain, contractile, e'
Obokata et al. [71]	Prospective single-center cohort	CHF, outpatient setting	HFpEF	≥ 50%	40	77 ± 13	65%		35%	88%		22 ± 5		LA reservoir strain, E/e', e', E/A
Carluccio et al. [72]	Prospective single-center observational study	CHF, outpatient setting	HFrEF	< 45%	747	68 ± 12	22%	16%	22%		48%	26 ± 4	32%	LAVi, E/e'
Gupta et al. [73]	Prospective on-going multi-comunities cohort	CHF, outpatient setting	HFpEF	≥ 50%	85	61.6 ± 6.9	85%		42%	85%	13%	32.6 ± 5.9	0%	E/A
			HFrEF	< 50%	31	60.9 ± 8.0	65%		68%	84%	32%	33.7 ± 9.6	10%	
Oh et al. [23]	International randomized trial	CHF, international (122 sites in 26 countries)	HFrEF	≤ 35%	2006	60.9 ± 9.5	13.6%	5%	37%	60%	99%		24%	LAVi, E/e', E/A, e'
Zile et al. [74]	Echo-cohort of placebo-controlled double-blind multi-center international parallel study	CHF, inpatient and outpatient settings	HFpEF (I-PRESERVE-Echo cohort)	≥ 45%	745	72 ± 7	62%	26%	25%	92%	33%	30 ± 5		E/e', LA area, e'
Tan et al. [75]	Prospective single-center cohort	CHF, outpatient setting	HFpEF	≥ 50%	50	72 ± 8	70%	excluded	30%	100%	18%	31 ± 5		LAVi
Jaubert et al. [76]	Prospective single-center cohort	CHF, inpatient setting	HFpEF	≥ 45%	59	64 ± 12	37%		36%	58%	49%	27 ± 5		LAVi, E/e', e'
Hinderliter et al. [77]	Prospective cohort	CHF, outpatient setting	HFrEF	≤ 40%	211	57 ± 12	31%	19%	44%	77%	43%	31.2 ± 7.2		LAVi
Donal et al. [78]	Prospective multi-center cohort	CHF, outpatient setting	HFrEF	< 35%	75	59 ± 11	82.7%				34.7%			LAVi
Jasic-Szpak et al. [79]	Prospective single-center cohort	CHF, outpatient setting	HFpEF without AF	≥ 50%	131	63.7 ± 8.0	73%	0	37%	89%	-	29.4 ± 4.1	-	LAVi, LA Reservoir, conduit and contractile strain, E/e', E/A
	Prospective single-center cohort	CHF, outpatient setting	HFpEF with AF	≥ 50%	39	67.4 ± 8.9	72%	100%	49%	97%	-	30.4 ± 4.3	-	
CarluccioE [80]	Prospective single-center cohort	CHF, outpatient setting	HFpEF	≥ 50%	46	75 ± 8	52%	-	35%	91%		-		LAVi, E/e', E/A

*ADHF* acute decompensated heart failure, *AHF* acute heart failure, *CHF* chronic heart failure, *CKD* chronic kidney disease, *HFpEF* heart failure with preserved ejection fraction, *HFrEF* heart failure with reduced ejection fraction, *LA* left atrial, *LAVi* left atrial volume index, *e’* mitral annular early diastolic velocity by tissue doppler, *E/A* the ratio between early and late mitral inflow velocity by doppler, *E/e’* the ratio between early mitral inflow velocity and mitral annular early diastolic velocity

**Table 3:** Echocardiographic characteristics of included studies

Author/year	HF phenotype	Number of patients (*n*)	LAVi	LAGLS_R_ (%)	LAGLS_B_ (%)	LAGLS_C_ (%)	E/e'	MV E/A	Mitral annulus e'	LVEF	LVGLS	Software for Speckle tracking analysis
Hoshida et al. [27]	HFpEF	105	47.6 ± 24.2				14.4 ± 5.7			60.9 ± 6.9		
Harada et al. [28]	HFpEF	92	54.6 ± 26,7				17.7 ± 7.3		5.2 ± 1.7	57.8 ± 9.4	−13.9 ± 4.4	EchoPAC
Hwang et al. [29]	HFpEF	1105	49.9(34.5–69.8)	18.6 ± 11.6			15.1 ± 7.1	0.9 ± 0.5	5.6 ± 2.5	59.3 ± 6.6	−15.1 ± 5.0	TomTec
Park et al. [16]	HFpEF	1191	63 ± 48.8	19.1 ± 11.6			16.9 ± 9.2		5.9 ± 2.4	59.1 ± 5.9		TomTec
Sobirin et al. [40]	HFpEF	30	33.0 ± 8.0				18.6 ± 3.4	1.09 ± 0.5	6.0 ± 1.6	56.0 ± 7.0		
Shah et al. [36]	HFpEF	1097	38.9 ± 15.5				12.6 ± 5.7	1.33 ± 0.73	7.9 ± 2.5	58.6 ± 9.8		
Reddy et al. [14]	HFpEF	238	32 ± 15	29 ± 16	18 ± 10	16 ± 9	14 ± 6				−15 ± 3	Syngo
Shintani et al. [37]	HFpEF	127	66.0 ± 27.4									
Wu et al. [38]	HFpEF	163	37.1 ± 8.1				16.5 ± 2.4	0.7 ± 0.1		68.0 ± 9.0		
Telles et al. [39]	HFpEF	49	41.5 ± 15.2	24.3 ± 9.6		16.1 ± 5.9	12.9 ± 5.7	1.4 ± 0.6	9.4 ± 2.4	62.6 ± 6.1	−18.7 ± 2.3	TomTec
Lundberg et al. [41]	HFpEF	92	43.0 ± 14.0	12.3 ± 8.0			13.4 ± 6.6	1.4 ± 0.9	8.5 ± 3.2	60.7 ± 5.9	−17.3 ± 4.4	EchoPAC
Shah et al. [50]	HFpEF-absent CMD	51	36.5 ± 11	19.8 ± 8.3			12.4 ± 4.7	1.2 ± 0.9	8.1 ± 2.4	60.9 ± 6.4	−17 ± 3.5	TomTec
	HFpEF-present CMD	151	39.3 ± 13.4	15.0 ± 7.7			13.5 ± 6.2	1.5 ± 0.9	8.9 ± 5.9	58.5 ± 8.1	−15.7 ± 3.5	TomTec
Abohammar et al. [53]	HFpEF	114	47.0 ± 7.0				12.2 ± 2.0	1.6 ± 0.7	7.0 ± 3.0	61.0 ± 3.0	−13.5 ± 1.5	EchoPAC
Saikhan et al. [42]	HFpEF	110	38.8 ± 12.7	26.2 ± 1.9	13.1 ± 4.4	13 ± 1.1	11.7 ± 4.5	0.9 ± 0.3	7.1 ± 2.1	64.9 ± 7.7		EchoPAC
Burns et al. [43]	HFpEF-Anemia	224	36.6 ± 15.8				16.1 ± 8.8	1.5 ± 0.8	9.6 ± 3.8	61.0 ± 7.0		
	HFpEF-No Anemia	195	31.3 ± 11.9				14.0 ± 7.3	1.2 ± 0.6	7.1 ± 2.7	61.0 ± 6.0		
Obokata et al. [44]	HFpEF	271	44 ± 15				16 ± 8		6.6 ± 2	62 ± 7		
Nagy et al. [45]	HFpEF	86	44 ± 16	13.3 ± 11.0			12.6 ± 6	1.8 ± 1.4	7.9 ± 2.2	62.5 ± 7.0	−15.3 ± 3.6	TomTec
Almeida et al. [48]	HFpEF	65	48.0 ± 19.4				16.0 ± 8.1			58.0 ± 5.9	−14.0 ± 3.7	EchoPAC
Liu et al. [49]	HFpEF	55	37.5 ± 8.3	20.4 ± 7.4	10.8 ± 4.2		12.8 ± 5.8	1.09 ± 0.73	7.7 ± 2.4	59.5 ± 6.5		EchoPAC
Batalli et al. [55]	HFpEF	55					9.4 ± 4.7	0.8 ± 0.3	6.7 ± 2.6	59.6 ± 8.7		Philips iE33
Sugimoto et al. [56]	HFpEF	20	52.0 ± 24.0	14.7 ± 7.4			20.0 ± 8.0	1.3 ± 0.9		56.0 ± 11.0		EchoPAC
Hage et al. [57]	HFpEF	86	44.4 ± 11.6				11.0 ± 4.2	1.5 ± 1.1	8.3 ± 2.2	63.3 ± 7.4		
Freed et al. [61]	HFpEF	308	34.4 ± 13.7	36.2 ± 14.9	18.3 ± 7.7	19.8 ± 8.5	15.0 ± 8.1	1.3 ± 0.7	7.0 ± 2.7	61.0 ± 6.4	− 7.5 ± 4.1	TomTec
Aung et al. [59]	HFpEF	38	43.7 ± 9.4	17.0 ± 4.1	11.0 ± 2.4		11.6 ± 1.8	0.7 ± 0.2	5.6 ± 1.5	62.9 ± 4.2		EchoPAC
Hunget al. [60]	HFpEF	58		28.2 ± 6.4			16.3 ± 6.3		5.9 ± 1.9	62.1 ± 6.3	−15.7 ± 1.8	EchoPAC
Unger et al. [62]	HFpEF-no CKD	154	32.5 ± 12.0	36.7 ± 16.3	19.1 ± 8.3	19.6 ± 8.9	11.8 ± 7.7	1.3 ± 0.7	10.1 ± 4.1	61.5 ± 6.3	−18.2 ± 4.0	TomTec
	HFpEF-CKD	145	36.5 ± 15.4	28.8 ± 14.9	15.9 ± 7.9	15.4 ± 7.2	14.8 ± 7.6	1.4 ± 0.7	8.5 ± 3.4	60.9 ± 6.6	−16.8 ± 4.1	TomTec
Shah et al. [66]	HFpEF	935	28.0 ± 10.3				10.9 ± 4.7	1.09 ± 0.53	7.6 ± 3.1	60.0 ± 6.4		
Melenovsky et al. [17]	HFpEF	101	41.0 ± 12.0						7.7 ± 2.2	62.0 ± 5.9		
Hasselberg et al. [65]	HFpEF	37	45.0 ± 22.0				11.0 ± 5.0	1.5 ± 1.1	7.1 ± 2.0	62.0 ± 7.0	−17.5 ± 3.2	EchoPAC
Gracia et al. [64]	HFpEF	28	32.6 ± 12.0				12.3 ± 3.6	1.0 ± 2.0	6.6 ± 1.4	65.0 ± 8.0		
Sanchis et al. [15]	HFpEF	63	58.9 ± 23.3		8.9 ± 4.9		11.3 ± 5.5			60.0 ± 5.0	−16 ± 3.7	EchoPAC
Santos et al. [67]	HFpEF	135	33.4 ± 11.5	24.6 ± 0.6			13.7 ± 8.6	1.2 ± 0.7	6.6 ± 2.4	59.0 ± 7.0	−15 ± 3.4	TomTec
Donal et al. [68]	HFpEF	539	49.4 ± 17.8				12.9 ± 6.1	1.8 ± 1.3	7.9 ± 2.6	62.0 ± 7.0	−19.0 ± 5.0	
Burker et al. [69]	HFpEF	419	34.2 ± 14.3				13.3 ± 7.9		9.3 ± 3.9	61.0 ± 7.0		
Obokata et al. [71]	HFpEF	40		22.7 ± 6.6	12.3 ± 5.9		19.8 ± 6.8	0.8 ± 0.3	3.3 ± 1.1	60 ± 13.3	−12.8 ± 3.5	EchoPAC
Gupta et al. [73]	HFpEF	85						1.0 ± 0.2				
Zile et al. [74]	HFpEF	746					10.0 ± 4.5	1.1 ± 0.7	9.1 ± 3.4	64.0 ± 9.0		
Tan et al. [75]	HFpEF	50	30.4 ± 9.2							62.0 ± 9.0		EchoPAC
Jaubert et al. [76]	HFpEF	59	30.7 ± 12.6				6.7 ± 2.7		10.8 ± 2.3			
Shah et al. [30]	HFrEF(recovered)	67	38.1 ± 12.5				22.4 ± 10.3	1.8 ± 1.0		26.4 ± 5.8		
	HFrEF (non-recovered)	69	47.1 ± 11.7				21.7 ± 8.8	2.0 ± 1.4		25.1 ± 7.1		
Tanaka et al. [31]	HFrEF	205	51.0 ± 20.0				14.3 ± 7.3			31.0 ± 8.0	−7.6 ± 2.0	Tomtec
Castrichini et al. [32]	HFrEF	77	57.0 ± 26.0	10.3 ± 6.9			16.7 ± 9.0			28.0 ± 6.0	−3 ± 4.0	Tomtec
Valentim et al. [33]	HFrEF	42	51.5 ± 22.6				13.6 ± 4.5			29.3 ± 6.4	−7.0 ± 2.6	
Deferm et al. [35]	HFrEF	31	69.0 ± 26.0	6.4 ± 2.2			16.8 ± 6.6	2.6 ± 0.7		20.0 ± 12.0	−7.3 ± 3.5	Tomtec
Park et al. [16]	HFrEF	2036	58.1 ± 28.8	11.7 ± 8.1			20.5 ± 11.9		4.7 ± 1.9	27.6 ± 7.3		Tomtec
Kurzawski et al. [34]	HFrEF	63	62.1 ± 13.3	8.9 ± 2.0			24.2 ± 8.4	2.4 ± 1.1		19.2 ± 4.1		EchoPAC
Modin et al. [18]	HFrEF	818	30.9 ± 13.8				12.2 ± 5.2	1.13 ± 0.67	6.9 ± 2.5	27.8 ± 9.1	−9.7 ± 3.3	EchoPAC
Shintani et al. [37]	HFrEF	617	67 ± 24.4									
Wu et al. [38]	HFrEF	34	38.4 ± 6.5				19.5 ± 7.7	1.28 ± 0.16		30 ± 9		
Lundberg et al. [41]	HFrEF	72	57.7 ± 18.5	7.7 ± 4.2			16.8 ± 9.0	2.8 ± 1.6	7.8 ± 2.9	28.3 ± 14.8	−7.2 ± 3.7	EchoPAC
Malagoli et al. [46]	HFrEF	286	46.2 ± 18.2	19.4 ± 9.4						31.6 ± 6.3		
Carluccio et al. [19]	HFrEF	405	52.6 ± 18.6	15.8 ± 7.0			14.3 ± 5.2	1.4 ± 1.2	5.4 ± 1.8	30.0 ± 7.4	−8.3 ± 2.9	EchoPAC
Eroglu et al. [47]	HFrEF	59	42.7 ± 22.1				17.0 ± 6.0	1.7 ± 1.7	5.3 ± 1.3	33.3 ± 10.4	−9.7 ± 4.4	Philips QLAB
Almeida et al. [48]	HFrEF	65	46.7 ± 13.3				17.7 ± 5.2			27.7 ± 11.9	−7.7 ± 2.2	EchoPAC
Xu et al. [51]	HFrEF-event	28	71.0 ± 22.0				19.3 ± 10.7	2.7 ± 0.8		17.0 ± 5.4		
	HFrEF-event-free	17	57.0 ± 16.0				20.5 ± 11.1	2.1 ± 1.2		19.0 ± 5.6		
Saha et al. [52]	HFrEF	49		11 ± 6			15 ± 10			31 ± 8	–7 ± 3	EchoPAC
Modin et al. [54]	HFrEF	151	42.1 ± 19.0				11.9 ± 5.3		8.6 ± 2.6	26.2 ± 9.4	−10.1 ± 3.6	EchoPAC
Batalli et al. [55]	HFrEF	56					13.5 ± 6.4	1.3 ± 0.9	5.3 ± 2.2	35 ± 7.5		
Sugimoto et al. [56]	HFrEF	49	55.0 ± 29.0	15.1 ± 10.1			24.0 ± 13.0	1.5 ± 1.1		31.0 ± 8.0		EchoPAC
Sargento et al. [58]	HFrEF	203	42.3 ± 18.3					1.4 ± 1.0		28.2 ± 8.4	−8.7 ± 3.3	EchoPAC
Melenovsky et al. [17]	HFrEF	97	50.0 ± 17.0						6.2 ± 2.1	24 ± 9.7		
Sanchis et al. [15]	HFrEF	32	57.8 ± 20.8		6.5 ± 5.4		11.6 ± 7.6			34.0 ± 10.0	−9.5 ± 4.5	
Motoki et al. [70]	HFrEF	108	42.0 ± 15.0	14.5 ± 8.2	7.7 ± 5.7		20.0 ± 12.0	1.7 ± 1.4	7.2 ± 4.5	25.0 ± 6.0		Syngo
Carluccio et al. [72]	HFrEF	747	43.9 ± 18.8				14.7 ± 8.0	1.77 ± 1.56	6.7 ± 2.8	29.0 ± 7.0		
Gupta et al. [73]	HFrEF	31						0.8 ± 0.3				
Oh et al. [23]	HFrEF	2006	41.9 ± 15.2				17.6 ± 9.6	1.3 ± 1.1	6.0 ± 3.0	28.9 ± 8.3		
Hinderliter et al. [77]	HFrEF	211	49 ± 23							32 ± 11		
Donal et al. [78]	HFrEF	75	43.4 ± 20.8									
Jasic-Szpak et al. [79]	HFpEF without AF	131	33.6 ± 9.3	29.0 ± 7.4	14.5 ± 4.0	14.4 ± 6.0	11.0 ± 2.8	0.87 ± 0.29	5.9 ± 1.2	72.7 ± 8.5	−18.6 ± 3.1	EchoPAC
	HFpEF with AF	39	39.9 ± 8.1	23.1 ± 6.5	10.9 ± 3.7	12.3 ± 4.9	13.6 ± 5.3	1.21 ± 0.78	5.5 ± 1.3	71.6 ± 8.5	−17.5 ± 3.8	
CarluccioE [80]	HFpEF	46	43.3 ± 16.9				16.7 ± 6.8	1.53 ± 0.87	5.9 ± 1.5	60 ± 6	−15.4 ± 3.5	EchoPAC

*HFpEF* heart failure with preserved ejection fraction, *HFrEF* heart failure with reduced ejection fraction, *HF* heart failure, *CMD* coronary microvascular dysfunction, *CKD* chronic kidney dysfunction, *GLS* global longitudinal strain, *LVGLS* left ventricle global longitudinal strain, *LVEF* left ventricle ejection fraction, *LA* left atrial, *LAVi* left atrial volume index, *LAGLS*_*R*_ left atrial global longitudinal strain at reservoir phase, *LAGLS*_*B*_ left atrial global longitudinal strain at booster phase, *LAGLS*_*C*_ left atrial global longitudinal strain at conduit phase, *e’* mitral annular early diastolic velocity by tissue doppler, *MV E/A* the ratio between early and late mitral inflow velocity by doppler, *E/e’* the ratio between early mitral inflow velocity and mitral annular early diastolic velocity

As compared to patients with HFrEF, patients with HFpEF appeared to be older, women, and had more often hypertension, AF and diabetes irrespective of inpatient or outpatient clinical setting (Table 1). The prevalence of IHD was 39.8% versus 30.7% in the acute inpatient setting and 49.8% versus 33.3% in the chronic outpatient setting when comparing patients with HFrEF versus HFpEF. Patients with HFrEF were more likely to be present with functional MR (27.2%) as compared to patients with HFpEF (12.0%) in the chronic ambulant setting of the study. The pooled mean value of BMI was 25.2 versus 25.6 kg/m^2^ in the acute inpatient setting and 27.5 versus 29.8 kg/m^2^ in the chronic outpatient in patients with HFrEF versus HFpEF. As expected by definition, patients with HFpEF had better LV systolic function as compared to patients with HFrEF with higher pooled LVEF and pooled absolute values of LVGLS irrespective of clinical setting of the study either acute inpatient or chronic outpatient (Table 1). Patients with HFpEF appeared to have higher pooled e’ (6.6 versus 7.5 cm/s in the acute inpatient versus chronic outpatient setting) than patients with HFrEF (4.7 versus 6.5 cm/s in the acute inpatient versus chronic outpatient setting). Conversely, the HFrEF group was characterized by higher E/e’ (18.5 versus 16.9 in the acute inpatient versus chronic outpatient setting) as compared to patients with HFpEF (14.0 versus 13.5 the acute inpatient versus chronic outpatient setting) irrespective of clinical setting of the study, indicating higher LV filling pressure in HFrEF.

### LA size and pressure estimated by LAVi and E/e’

Twenty-nine studies reported LAVi in patients with HFrEF (*n* = 8726), and thirty-eight studies reported LAVi in patients with HFpEF (*n* = 9049). The pooled mean value of LAVi was 59.7 versus 48.3 ml/m^2^ in the acute inpatient versus chronic outpatient setting for patients with HFrEF, and 52.7 versus 38.2 ml/m^2^ in the acute inpatient versus chronic outpatient setting for patients with HFpEF. Eight out of 41 included studies reported LAVi in both patients with HFrEF (*n* = 3002) and HFpEF (*n* = 1822). In these eight studies, LAVi was comparable between patients with HFrEF and HFpEF [pooled mean LAVi, 42.7 versus 37.6 ml/m^2^; weighed mean difference [WMD] = −0.2 (−0.48, 0.07); *p* = 0.15; *I*^2^ = 89.8%]. Three out of these eight studies enrolled both patients with HFrEF (*n* = 2718) and HFpEF (*n* = 1383) in the acute hospitalized setting, where the remaining studies included patients with both HF phenotypes in the chronic stable setting (HFrEF, *n* = 284; HFpEF, *n* = 439). In both acute inpatient [pooled mean LAVi, 54.8 versus 52.6 ml/m^2^ in HFrEF versus HFpEF; WMD = −0.2 (−0.48, 0.07); *p* = 0.13; *I*^2^ = 89.8%] and outpatient setting [pooled mean LAVi, 42.7 versus 36.9 ml/m^2^ in HFrEF versus HFpEF; WMD = −0.2 (−0.48, 0.07); *p* = 0.153; *I*^2^ = 89.8%], the LAVi was comparable between patient with HFrEF and HFpEF, although the difference between HFrEF and HFpEF patients appeared to be more narrowed in acute inpatient HF settings. Seven out of 41 included studies reported E/e’ in both patients with HFrEF and HFpEF (HFrEF, *n* = 2344; HFpEF, *n* = 1649). In these studies, E/e’ was significantly higher in patients with HFrEF as compared to patients with HFpEF [15.9 versus 13.4 in HFrEF versus HFpEF; WMD = −0.40 (−0.56, −0.24); *p* < 0.05, *I*^2^ = 77.6%]. However, in the acute inpatient setting, E/e’ was comparable between patients with HFrEF and HFpEF [17.7 versus 14.0 in HFrEF versus HFpEF; WMD = −0.40 (−0.56, −0.24); *p* = 0.15, *I*^2^ = 77.6%], whereas E/e’ was significantly higher in patients with HFrEF as compared to patients with HFpEF in chronic HF setting [15.3 versus 13.3 in HFrEF versus HFpEF; WMD = − 0.40 (−0.56, −0.24); *p* < 0.05, *I*^2^ = 77.6%].

### LA function estimated by LA reservoir, booster, and conduit GLS

**Fig. 2:**
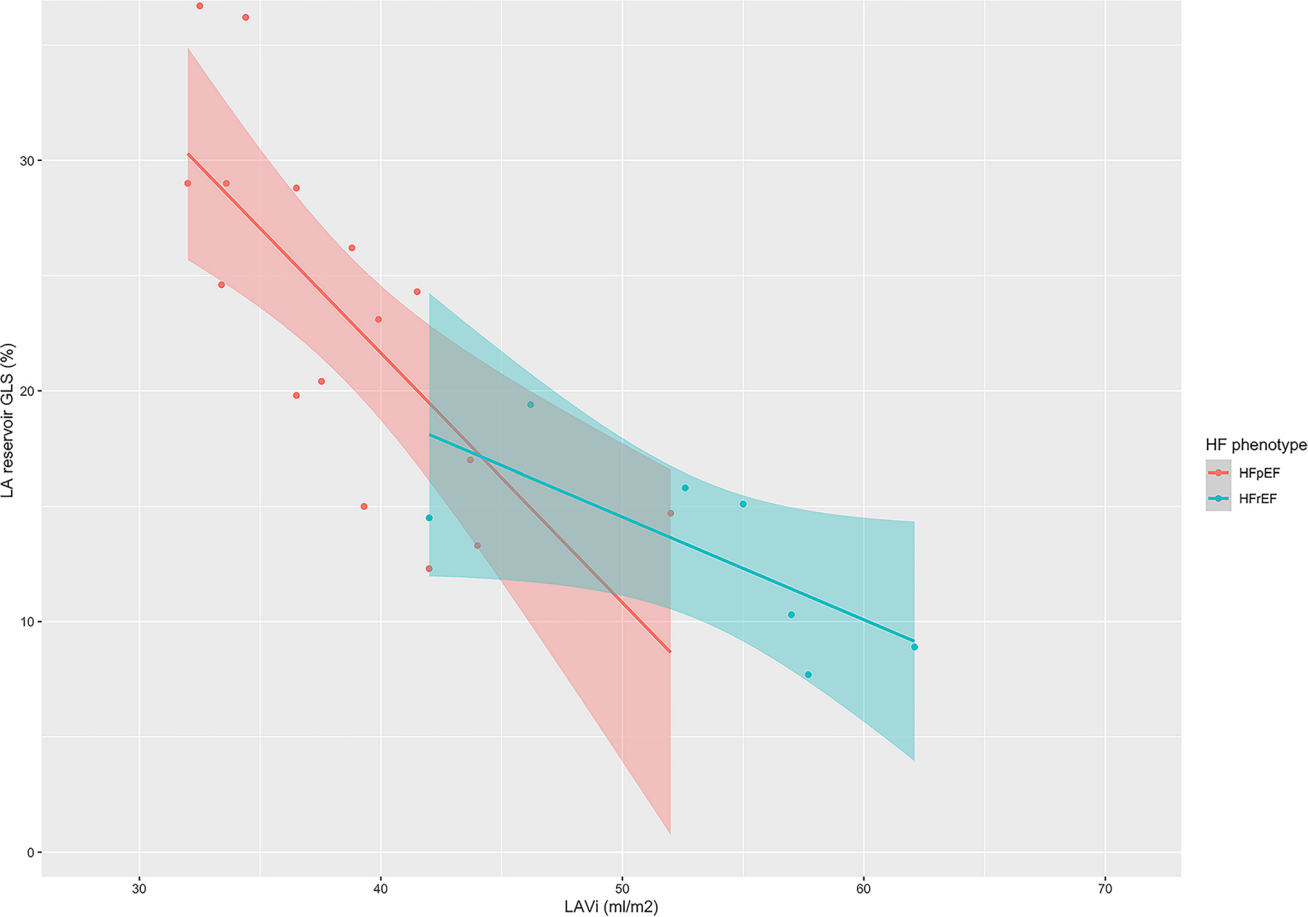
Meta-analytic scatterplot for the relationship between LAVi and LA reservoir GLS in patients with HFpEF versus HFrEF. HFpEF, heart failure with preserved ejection fraction; HFrEF, heart failure with reduced ejection fraction; LAVi, left atrial volume index; LA, left atrial; GLS, global longitudinal strain

Ten studies reported LA reservoir GLS (LAGLS_R_) in patients with HFrEF (*n* = 3176), and seventeen studies reported LAGLS_R_ in patients with HFpEF (*n* = 4196). The pooled mean value of LAGLS_R_ was 9.0 versus 12.8% in the acute inpatient versus chronic outpatient setting for patients with HFrEF, and 18.9 versus 23.4% in the acute inpatient versus chronic outpatient setting for HFpEF patients. Four out of 61 studies in the chronic outpatient setting reported LAGLS_R_ in both patients with HFpEF (*n* = 1877) and HFrEF (*n* = 3058). LAGLS_R_ was worse in patients with HFrEF as compared to patients with HFpEF [8.5% versus 23.6%; WMD = 16.3% (22.05, 8.61); *p* < 0.001, *I*^2^ = 77.6%]. Besides, the relationship between LAVi and LAGLS_R_ (Fig. 2) was significant in HFpEF (estimated coefficient −1.08, *p* = 0.009, *R*^2^ = 0.525), but not in HFrEF (estimated coefficient −0.44, *p* = 0.06, *R*^2^ = 0.447). On the other hand, the relationship between LAGLS with LVGLS was not significant in neither HFpEF (estimated coefficient 1.35, *p* = 0.30, *R*^2^ = 0.01) nor HFrEF (estimated coefficient 2.81, *p* = 0.41, *R*^2^ = 0.006). Two studies reported LA booster GLS (LAGLS_B_) in patients with HFrEF (*n* = 140), and ten studies reported LAGLS_B_ in patients with HFpEF (*n* = 1320). The pooled mean value of LAGLS_B_ was 7.7% versus 13.9% between patients with HFrEF and HFpEF in the chronic ambulant clinical setting. None of the included studies reported the LAGLS_B_ in both patients with HFpEF and HFrEF. Five studies reported LA conduit GLS (LAGLS_C_) in patients with HFpEF (*n* = 1173) in the chronic ambulant clinical setting, and the pooled mean value LAGLS_C_ was 15.8% in patients with HFpEF. No included studies reported LAGLS_C_ in patients with HFrEF. Given the very limited number of studies comparing LA booster and conduit function in patients with HFrEF versus HFpEF, it is hard to determine how these two LA phasic function differ in patients with HFrEF versus HFpEF. Lastly, the details of prognostic information for each LA parameter and the adjusted covariates from included studies were summarized in supplementary online (Tables S5 and S6).

## Discussion

To the best of our knowledge, this is the first systematic review and meta-analysis assessing and comparing LA structural and functional echocardiographic parameters and their clinical relevance in patients with HFrEF versus HFpEF. It comprehensively summarized 61 studies, among which 27 studies with HFrEF patients (*n* = 8806) and 38 studies with HFpEF patients (*n* = 9928). Several important clinical findings emerged from the current study:

(1) LA volumes were comparable between patients with HFrEF and HFpEF; (2) LV filling pressures (estimated by E/e’) were comparable between patients with HFrEF and HFpEF in the acute inpatients setting, while in the chronic outpatient setting, LV filling pressures were higher in patients with HFrEF; (3) the LA reservoir GLS was profoundly lower in patients with HFrEF as compared to patients with HFpEF, despite the greater burden of AF in patients with HFpEF and clinical setting of the study (acute inpatient or chronic outpatient).

The left atrium is an easily expandable thin-walled structure that plays a crucial role in LV filling and optimizing cardiac output through interaction with both LV and pulmonary veins through the entire cardiac cycle [1]. It possesses three main functions, including mechanical, endocrine, and regulatory functions, which are closely intertwined and tightly coupled with one another [1]. Rapid development and application of 2D strain to the LA have enabled us to better understand the mechanical function of LA, which is composed of the reservoir, conduit, and booster functions based on the corresponding LA phase in the cardiac cycle [1]. Furthermore, a recent meta-analysis reported the normal values of each strain component, with LA reservoir, conduit, and booster GLS as 39%, 23%, and 17%, respectively [13]. Based on these reference values it can be concluded that pronounced LA dysfunction exists in both patients with HFpEF and HFrEF, further supporting the concept of LA myopathy. Most interestingly, it was recently described that LA reservoir GLS outperformed E/e’ and LAVi in the diagnosis of the HFpEF [14].

Several studies have compared LA structure and function using various imaging methods with mixed results in patients with HFrEF versus HFpEF, which was the starting point of our systematic review. For example, Sanchis et al. showed that LAVi and LA longitudinal strain were similar in new-onset outpatients with HFrEF versus HFpEF [15]. In contrast, LA dysfunction (using LAGLS) was worse in acute heart failure patients with HFrEF than HFpEF, but equally associated with survival [16]. Melenovsky et al. used LA ejection fraction (LAEF) and showed that LA dysfunction was associated with mortality only in patients with HFpEF despite worse LA function in patients with HFrEF [17]. In contrast, Modin et al. showed LAEF was independently associated with mortality in a larger sample of HFrEF patients [18], and Carluccio et al. showed that LA reservoir GLS was independently associated with survival in a cohort of patients with HFrEF [19]. Finally, a recent meta-analysis, pooling data of HFpEF studies, showed that LA reservoir strain was associated with prognosis in patients with HFpEF [5].

A change in LA structure and function is a complex, dynamic and heterogeneous process that may be different between phenotypes of HF. LA dysfunction and increase of LA pressure have long been considered as hallmarks of HFpEF, whereas HFrEF is generally considered as a left ventricular disease [3, 20, 21]. This might explain the discrepancy in the number of studies focusing on LA dysfunction in HFpEF versus HFrEF. However, despite a greater burden of AF in patients with HFpEF, our data found that LA function was worse in patients with HFrEF than patients with HFpEF. This might be related to the greater burden of moderate to severe functional MR in patients with HFrEF. HFrEF is more associated with an eccentric ventricular remodelling, resulting in tethering of the mitral leaflets [22, 23]. In our review, we showed that in HFpEF patients functional MR was less prevalent, but not negligible, and may be more the result of mitral annular dilation due to the high incidence of AF in this subgroup.

LA reservoir peak longitudinal strain, inherent to its nature as a strain, is dependent on its baseline length, with maximal elongation of the LA during LV systole, suggesting its high dependence on LV longitudinal strain as well [24]. Carluccio et al. showed that LA reservoir GLS was more strongly associated with LVGLS beyond LA volume and E/e’ in patients with HFrEF, supporting the significant contribution of LV systolic dysfunction to LA dysfunction in patients with HFrEF [19]. Comparatively, LA mechanical dysfunction in patients with HFpEF, particularly in the setting of AF, is usually not accompanied by substantial changes of LV systolic function, which suggests LA mechanical dysfunction to be disproportionate to LV systolic dysfunction in such patients [8]. Hence, a decrease of LV longitudinal function, as we show in patients with HFrEF, might impact LA reservoir function more in patients with HFrEF than HFpEF [17, 20], suggesting that the concept of LA myopathy is not only subject to HFpEF, but to HFrEF as well.

Despite worse LA global function in HFrEF than HFpEF, the prevalence of AF was higher in patients with HFpEF than HFrEF. AF and HFpEF share many convergent metabolic risk factors, including obesity that promote systematic inflammatory processes. Expansion of epicardial fat tissue may act as a local source of inflammation, amplifying ongoing systemic inflammatory processes [20]. LA dysfunction in HFpEF is likely associated with a series of inflammatory cascades resulting in coupled LA endocrine and regulatory dysfunctions. This is supported by data from Patel et al. who showed that LA reservoir strain was associated with biomarkers of neurohormonal activation [25]. However, the exact mechanism of how the LA mechanical, regulatory, and endocrine functions are coupled together, and particular which factor is the main driving component of LA dysfunction in both settings of HFpEF and HFrEF remains unknown.

Although the prognostic value of LA reservoir strain has been described in several studies that were included in our systematic review both in patients with HFpEF and HFrEF [16, 18, 19], future prognostic studies are warranted to investigate whether LA dysfunction in HFrEF and HFpEF are two distinct processes. A better understanding of different forms of LA dysfunction in HFrEF versus HFpEF may have important clinical implications. Given the distinct LA reservoir GLS in patients with HFrEF versus HFpEF, this measurement might serve as a potential marker to better phenotype patients with HF. For patients with HFpEF, a novel therapeutic intervention which specifically targets the LA by creating a shunt at the atrial level to offload LA pressure looks promising from preliminary data [26]. Given our finding of higher LA pressure and worse LAGLS in HFrEF, we might cautiously postulate a potential benefit of this novel device in patients with HFrEF as well.

## Limitations

There are several limitations of the current systematic review. First, our review has the inherent limitation of selection and reporting bias, which was minimized by a thorough selection procedure and quality assessment. Secondly, we only focused on primary echocardiographic parameters assessing LA structures and function that have been widely recommended in guidelines. Other echocardiographic parameters such as LAEF and other LA-related parameters assessed by other imaging modalities were not included in the current review. Thirdly, we were not able to account for all differences in clinical characteristics due to a lack of individual-level data. For example, the definition (and thus the extent) of ischemic cardiomyopathy varies study by study, which hampers a thorough analysis of its (possibly) confounding role. Fourth, we were unable to report the weighted HR of comprehensive LA structural and functional parameters except for LA reservoir GLS due to the limited numbers of studies, different outcome measures, and lack of confounder adjustments. Last but not least, the details of averaging the RR interval for the strain measurement in the setting of AF were not addressed in most of the studies.

## Conclusion

Although left atrial abnormalities have been proposed as a hallmark of HFpEF, we found that LA structure and function are worse in patients with HFrEF than HFpEF. Thus, the significant pathophysiological insight of intrinsic LA myopathy should be equally emphasized in both patients with HFrEF and patients with HFpEF.

## Supplementary Information

Below is the link to the electronic supplementary material.

Supplementary file1 (DOCX 54 KB)
