# Supplementary material for: Left atrial structure and function in heart failure with reduced (HFrEF) versus preserved ejection fraction (HFpEF): systematic review and meta-analysis
Source: Heart Fail Rev. 2022 Jan 26;27(5):1933–55. doi: 10.1007/s10741-021-10204-8 (PMC9388424; doi:10.1007/s10741-021-10204-8)
Supplement: Supplementary file 1 — Supplementary file1 (DOCX 54 KB) [file 10741_2021_10204_MOESM1_ESM.docx]

SUPPLEMENTARY MATERIAL

**Table S1 Detailed search strategy**

| MEDLINE search strategy  ("heart failure"[tiab] OR “heart failure with preserved ejection fraction”[tiab] OR “heart failure with reduced ejection fraction”[tiab] OR "HFREF”[tiab] OR “HFPEF”[tiab] OR “HFNLEF”[tiab] OR “HFNEF”[tiab])  AND (("left atrial"[tiab] OR “left atrium”[tiab] OR “atrial”[tiab]) AND (“structure”[tiab] OR “size”[tiab] OR “enlargement”[tiab] OR “volume”[tiab] OR "lavi"[tiab] OR “LA volume”[tiab]) AND (echocar*[tiab] OR ultraso*[tiab]))  AND (("left atrial"[tiab] OR “left atrium”[tiab] OR “atrial”[tiab]) AND (“strain”[tiab] OR “deformation”[tiab] OR “function”[tiab] OR “speckle tracking”[tiab] OR “STE”[tiab] OR “phasic function”[tiab] OR “stiffness”[tiab]))  EMBASE search strategy  ('heart failure with reduced ejection fraction'/exp OR 'heart failure with reduced ejection fraction' OR 'heart failure with preserved ejection fraction'/exp OR 'heart failure with preserved ejection fraction' OR 'diastolic heart failure'/exp OR 'diastolic heart failure' OR 'systolic heart failure'/exp OR 'systolic heart failure' OR 'heart failure'/exp OR 'heart failure' OR 'congestive heart failure'/exp OR 'congestive heart failure' OR 'acute heart failure'/exp OR 'acute heart failure' OR 'heart failure with normal ejection fraction'/exp OR 'heart failure with normal ejection fraction' OR 'heart failure with reduced left ventricular ejection fraction'/exp OR 'heart failure with reduced left ventricular ejection fraction' OR 'heart failure with preserved left ventricular ejection fraction'/exp OR 'heart failure with preserved left ventricular ejection fraction')  AND ('left atrial volume index'/exp OR 'left atrial volume index' OR 'left atrial volume'/exp OR 'left atrial volume' OR 'left atrial enlargement'/exp OR 'left atrial enlargement')  AND ('left atrial strain'/exp OR 'left atrial strain' OR 'left atrial dysfunction'/exp OR 'left atrial dysfunction' OR 'speckle tracking echocardiography'/exp OR 'speckle tracking echocardiography' OR 'speckle tracking imaging'/exp OR 'speckle tracking imaging') |
| --- |

**Table S2. Quality assessment of observational studies**

|  | **Selection** | | | **Comparability** | **Outcome** | |
| --- | --- | --- | --- | --- | --- | --- |
|  | **S1** | **S2** | **S3** | **C** | **O1** | **O2** |
| Hoshida^1^ 2020 | * | * | ** |  | ** | * |
| Harada^2^ 2020 | * | * | ** |  | ** | * |
| Hwang^3^ 2020 | * | * | ** |  | ** | * |
| Shah^4^ 2020 | * | * | ** | * | ** | * |
| Tanaka^5^ 2020 | * | * | ** |  | ** | * |
| Castrichini^6^ 2020 | * | * | ** |  | ** | * |
| Valentim^7^ 2020 | * | * | ** | * | ** | * |
| Kurzawski^8^ 2020 | * | * | ** |  | ** | * |
| Park^9^ 2020 | * | * | ** | * | ** | * |
| Deferm^10^ 2019 | * | * | ** |  | ** | * |
| Reddy^11^ 2019 | * | * | ** |  | ** | * |
| Modin^12^ 2019 | * | * | ** |  | ** | * |
| Shintani^13^2019 | * | * | ** | * | ** | * |
| Wu^14^2019 | * | * | ** | * | ** | * |
| Telles^15^ 2019 | * | * | ** |  | ** | * |
| Sobirin^16^ 2019 | * | * | ** |  | ** | * |
| Lundberg^17^ 2018 | * | * | ** | * | ** | * |
| Saikhan^18^ 2018 | * | * | ** | * | ** | * |
| Burns^19^ 2018 | * | * | ** |  | ** | * |
| Obokata^20^ 2018 | * | * | ** |  | ** | * |
| Nagy^21^ 2018 | * | * | ** |  | ** | * |
| Carluccio^22^ 2018 | * | * | ** |  | ** | * |
| [Malagoli^23^2018](https://www.ncbi.nlm.nih.gov/pubmed/?term=Malagoli%20A%5BAuthor%5D&cauthor=true&cauthor_uid=30316541) | * | * | ** |  | ** | * |
| Eroglu^24^ 2018 | * | * | ** | * | ** | * |
| Almeida^25^ 2018 | * | * | ** | * | ** | * |
| Liu^26^ 2018 | * | * | ** | * | ** | * |
| Shah^27^ 2018 | * | * | ** | * | ** | * |
| Xu^28^ 2018 | * | * | ** |  | ** | * |
| Saha^29^2018 | * | * | ** |  | ** | * |
| Abohammar^30^ 2018 | * | * | ** |  | ** | * |
| Modin^31^ 2018 | * | * | ** |  | ** | * |
| Batalli^32^ 2017 |  | * | ** | * | ** | * |
| Sugimoto^33^ 2017 | * | * | ** | * | ** | * |
| Hage^34^ 2017 | * | * | ** |  | ** | * |
| Sargento^35^ 2017 | * | * | ** |  | ** | * |
| Aung^36^ 2016 | * | * | ** | * | ** | * |
| Hung^37^2016 | * | * | ** |  | ** | * |
| Freed^38^2016 | * | * | ** |  | ** | * |
| Unger^39^ 2016 | * | * | ** | * | ** | * |
| Georgievska-Ismail^40^ 2016 | * | * | ** | * | ** | * |
| Melenovsky^41^ 2015 | * | * | ** | * | ** | * |
| Gracia^42^ 2015 | * | * | ** | * | ** | * |
| [Hasselberg^43^2015](https://www.ncbi.nlm.nih.gov/pubmed/?term=Hasselberg%20NE%5BAuthor%5D&cauthor=true&cauthor_uid=25552469) | * | * | ** | * | ** | * |
| Sanchis^44^ 2015 | * | * | ** | * | ** | * |
| Donal^45^ 2014 | * | * | ** |  | ** | * |
| Burke^46^2014 | * | * | ** |  | ** | * |
| Motoki^47^ 2013 | * | * | ** |  | ** | * |
| Obokata^48^ 2013 | * | * | ** | * | ** | * |
| Carluccio^49^ 2013 | * | * | ** |  | ** | * |
| Gupta^59^ 2013 | * | * | ** | * | ** | * |
| Tan^50^ 2010 | * | * | ** | * | ** | * |
| Jaubert^51^ 2010 | * | * | ** |  | ** | * |
| Hinderliter^52^ 2008 | * | * | ** |  | ** | * |
| Donal^53^ 2008 | * | * | ** |  | ** | * |
| Jasic-Szpak^60^ 2021 | * | * | ** | * | ** | * |
| Carluccio E^61^ 2016 | * | * | ** | * | ** | * |

**Table S3. Revised Cochrane risk-of-bias tool for randomized trials (RoB 2.0)**

| Study | Randomization | Intervention | Missing Data | Outcome Measurement | Reported Results | Overall Risk |
| --- | --- | --- | --- | --- | --- | --- |
| Shah^54^2019 | Low | Low | Low | Low | Low | Low |
| Shah^55^2014 | Low | Low | Low | Low | Low | Low |
| Santos^56^2014 | Low | Low | Low | Low | Low | Low |
| Oh^57^2012 | Low | Low | Low | Low | Low | Low |
| Zile^58^ 2011 | Low | Low | Some concerns | Low | Low | Some concerns |

**Table S4 Reason of exclusion of papers**

| **Reason** |  |
| --- | --- |
| No HF study population | 498 |
| Case reports, review, editorial comment etc, not original research | 172 |
| Selected HFpEF or HFrEF group, ie HCM, TCM, Amyloidosis or CRT or any kinds of interventions | 197 |
| No LA related echocardiographic parameters reported | 165 |
| Cardiac functional assessments from other imaging modalities, and 3D echocardiography | 36 |
| Animal study | 40 |
| Not in adults | 10 |
| Not in English | 3 |
| Combined HFpEF, HFrEF study population, HFmrEF | 5 |
| Duplicate | 23 |
| Insufficient related information from solely abstract | 4 |
| **Total** | **1153** |

**Table S5. Prognostic characteristics related to LA parameters**

| **Study/Publication year** | **HF phenotype** | **Follow-up months** | **Outcome measure** | **LA echo measure** | **Unadjusted HR (95%CI)** | **Adjusted HR (95%CI)** |
| --- | --- | --- | --- | --- | --- | --- |
| Park^9^ 2020 | HFpEF | 30.6 months (11.6-54.4 months) | All-cause mortality/hospitalization | LA reservoir GLS | 0.98(0.97-0.98), P<0.001 | NA |
| Nagy^21^ 2018 | HFpEF | 572 days | All-cause mortality | LA reservoir GLS<20% | 3.23(1.1-9.3), *p*=0.029 | 4.15 (1.2-14), *p*=0.023 |
| Freed^38^ 2016 | HFpEF | 13.8 (4.5-23.9) months | CV outcomes | LA reservoir GLS | 1.72(1.32-2.15), *p*<0.001 | 1.43 (1.05-1.95), *p*=0.02 |
| Burker^46^ 2014 | HFpEF | 17.7 (9.5-30) months | CV outcomes | LAVi (per increasing 1ml/m^2^) | 1.16(1.02-1.33), *p*=0.03 | 1.02(0.85-1.21), *p*=0.85 |
| Zile^58^ 2011 | HFpEF | 49.5 months | All-cause mortality/CV hospitalization | LA area enlargement | 3.52(2.00-6.19), *p*<0.001 | 2.26 (1.24-4.12), *p*=0.008 |
| Park^9^ 2020 | HFrEF | 30.6 months (11.6-54.4 months) | All-cause mortality/hospitalization | LA reservoir GLS | 0.97(0.96-0.98), *p*<0.001 | NA |
| [Malagoli^23^2019](https://www.ncbi.nlm.nih.gov/pubmed/?term=Malagoli%20A%5BAuthor%5D&cauthor=true&cauthor_uid=30316541) | HFrEF | 48±11 months | CV mortality/MACE | LA reservoir GLS | 0.94(0.90-0.97), *p*<0.001 | 0.94 (0.92-0.95), *p*<0.01 |
| Carluccio^22^2018 | HFrEF | 30 months (13.1-51.3) | All-cause mortality/ HF hospitalization | LA reservoir GLS | 1.97(1.32-2.09), *p*<0.0001 | 1.38 (1.03-1.84), *p*=0.03 |
| Modin^12^ 2019 | HFrEF | 3.3years (1.8-4.6) | All-cause mortality | LAVi (per increasing 5ml/m^2^) | 1.14(1.09-1.19), *p*<0.001 | 1.10 (1.05-1.16), *p*<0.001 |
| Xu^28^ 2018 | HFrEF | 1 year | Cardiac mortality/LV assist device insertion within 1 year | LAVi (per increasing 1ml/m^2^) | 1.02(1.00-1.04), *p*=0.049 | NA |
| Modin^31^ 2018 | HFrEF | 2.7 years (median follow-up) | All-cause mortality | LAVi (per increasing 1ml/m^2^) | 1.02(1.00-1.03), *p=*0.036 | 1.02 (1.00-1.03), *p=*0.08 |
| Carluccio^49^ 2013 | HFrEF | 34±23 months | All-cause mortality | LAVi (per increasing 15ml/m^2^) | 1.49(1.36-1.65), *p<*0.01 | 1.38 (1.20-1.59), *p<*0.01 |
| Hinderlite^52^ 2008 | HFrEF | 4 years (3-6) | All-cause mortality/ CV death | LAVi (per increasing 1ml/m^2^) | 1.66(1.41-1.95), *p*<0.001 | 1.38(1.1-1.72), *p*=0.005 |

Abbreviations: CV: cardiovascular; LA: Left Atrial; LAVi: left atrial volume index; GLS: global longitudinal strain; HFpEF: Heart Failure with Preserved Ejection Fraction; HFrEF: Heart failure with reduced ejection fraction

**Table S6 Adjustment variables in prognostic studies**

| Study/year | LA related variable | Adjustment variables |
| --- | --- | --- |
| Park^9^ 2020 | LA reservoir GLS | Only univariate analysis |
| Shah^54^2019 | E/e’, LAVi | Age, sex, region of enrollment, and randomized treatment assignment, NT-proBNP, hypertension, AF, prior HF hospitalization, prior myocardial infarction, diabetes, NYHA functional class, and mineral corticoid receptor antagonist use |
| Modin^12^ 2019 | LAVi | Age, sex, diabetes, mean arterial pressure, treatment with diuretics and atrial fibrillation |
| [Malagoli^23^2019](https://www.ncbi.nlm.nih.gov/pubmed/?term=Malagoli%20A%5BAuthor%5D&cauthor=true&cauthor_uid=30316541) | LA reservoir GLS | Age, NYHA functional class, GFR, BNP, LVESVi, LAVi_max_, LVEF, E/A ratio, E/e’ |
| Carluccio^22^ 2018 | LA reservoir GLS | EMPHASIS-HF risk score (which includes the following variables: age, sex, systolic blood pressure, estimated GFR, diabetes mellitus, prior HF hospitalization, hemoglobin, prior myocardial infarction/coronary artery bypass graft, BMI, and heart rate), NYHA class, log BNP, ICD at baseline, and CRT implant during follow-up, LAVi, LVEDVI, EF, E/E’ ratio, and mitral regurgitation severity |
| Nagy^21^ 2018 | LA reservoir GLS | Age, eGFR, LVGLS, Tau index |
| Modin^31^ 2018 | LAVi | Age, sex, MAP, and heart rate |
| Freed^38^ 2016 | LA reservoir GLS | Sex, atrial fibrillation, MAGGIC risk score (which includes the following variables: age, LVEF, creatinine, diabetes, chronic obstructive pulmonary disease, systolic blood pressure, body-mass index, heart rate, NYHA class, ACE-inhibitor use, beta-blocker use, heart failure duration, and current smoker); LV mass; and LA volume, E/e’ ratio, LV longitudinal strain and RV free wall strain. |
| Burke^46^ 2014 | LAVi, E/e’ | Age, sex, BMI, coronary artery disease, diabetes mellitus, atrial fibrillation,  chronic obstructive pulmonary disease, obstructive sleep apnea, hypertension, estimated GFR, hemoglobin, degree  of mitral regurgitation, LV mass index, and NYHA functional class |
| Carluccio^49^ 2013 | LAVi | LAVi, LVESVi, DT, PASP, TAPSE |
| Zile^52^ 2011 | LA area enlargement | Log NT-proBNP, age, diabetes, hospitalization for worsening heart failure within 6 months preceding randomization, COPD or asthma, neutrophils, and LVEF. |
| Hinderliter^58^  2008 | LAVi | Age, NT-proBNP, LVEF, LVEDV, tricuspid regurgitation area, left atrial and right atrial volume |

Abbreviations: AF: atrial fibrillation; BMI: Body Mass Index; BNP: natriuretic peptide; NT-proBNP: N-terminal pro-B type natriuretic peptide; CRT: cardiac resynchronization therapy; COPD: chronic obstructive pulmonary disease; DT: deceleration time; GFR: glomerular filtration rate; eGFR: estimated glomerular filtration rate; ICD: Implantable cardioverter defibrillator; LA: Left Atrial; LAVi: left atrial volume index; LVESVi: left ventricular end-systole volume index; LVEF: left ventricular ejection fraction; LVEDV: left ventricular end-diastole volume; LVEDVi: end-diastole volume index; LVGLS: left ventricle global longitudinal strain; GLS: global longitudinal strain; HF: heart failure; HFpEF: HF with Preserved Ejection Fraction; HFrEF: HF with reduced ejection fraction; MAP: mean atrial pressure; NYHA: New York Heart Association; RV: right ventricular; PASP: pulmonary arterial systolic pressure; TAPSE: tricuspid annular plane systolic excursion.

**Reference**

1. Hoshida S, Watanabe T, Shinoda Y, Minamisaka T, Fukuoka H, Inui H, Ueno K, Yamada T, Uematsu M, Yasumura Y, Nakatani D, Suna S, Hikoso S, Higuchi Y, Sakata Y, Osaka CardioVascular Conference I. Considerable scatter in the relationship between left atrial volume and pressure in heart failure with preserved left ventricular ejection fraction. *Sci Rep*. 2020;**10**(1):90.
2. Harada T., Sunaga H., Sorimachi H., et al. Pathophysiological role of fatty acid‐binding protein 4 in Asian patients with heart failure and preserved ejection fraction. ESC Hear Fail 2020;**7**(6):4256-4266.
3. Hwang IC., Cho GY., Choi HM., et al. H2FPEF Score Reflects the Left Atrial Strain and Predicts Prognosis in Patients With Heart Failure With Preserved Ejection Fraction. J Card Fail 2020;**S1071-9164**(20):31464-0.
4. Shah MA., Soofi MA., Jafary Z., et al. Echocardiographic parameters associated with recovery in heart failure with reduced ejection fraction. Echocardiography 2020;**37**(10):1574-1582.
5. Tanaka H., Tatsumi K., Matsuzoe H., Matsumoto K., Hirata KI. Impact of diabetes mellitus on left ventricular longitudinal function of patients with non-ischemic dilated cardiomyopathy. Cardiovasc Diabetol 2020;**19**(1):84
6. Castrichini M., Manca P., Nuzzi V., et al. Sacubitril/Valsartan Induces Global Cardiac Reverse Remodeling in Long-Lasting Heart Failure with Reduced Ejection Fraction: Standard and Advanced Echocardiographic Evidences. J Clin Med 2020;**9**(4):906.
7. Valentim Gonçalves A., Galrinho A., Pereira-Da-Silva T., et al. Myocardial work improvement after sacubitril-valsartan therapy: A new echocardiographic parameter for a new treatment. J Cardiovasc Med 2020;**21**(3):223-230.
8. Kurzawski J, Janion-Sadowska A, Zandecki L, Piatek L, Koziel D, Sadowski M. Global peak left atrial longitudinal strain assessed by transthoracic echocardiography is a good predictor of left atrial appendage thrombus in patients in sinus rhythm with heart failure and very low ejection fraction - an observational study. Cardiovasc Ultrasound. 2020;**18**(1):7.
9. Park JH, Hwang IC, Park JJ, Park JB, Cho GY. Prognostic power of left atrial strain in patients with acute heart failure. *Eur Heart J Cardiovasc Imaging*. 2021;**2**(2):210-219.
10. Deferm S, Martens P, Verbrugge FH, Bertrand PB, Dauw J, Verhaert D, Dupont M, Vandervoort PM, Mullens W. LA Mechanics in Decompensated Heart Failure: Insights From Strain Echocardiography With Invasive Hemodynamics. *JACC Cardiovasc Imaging*. 2020;S1936-878X(19)31178-7.
11. Reddy YNV, Obokata M, Egbe A, Yang JH, Pislaru S, Lin G, Carter R, Borlaug BA. Left atrial strain and compliance in the diagnostic evaluation of heart failure with preserved ejection fraction. *Eur J Heart Fail*. 2019;**21**(7):891-900.
12. Modin D, Sengelov M, Jorgensen PG, Olsen FJ, Bruun NE, Fritz-Hansen T, Andersen DM, Jensen JS, Biering-Sorensen T. Prognostic Value of Left Atrial Functional Measures in Heart Failure With Reduced Ejection Fraction. *J Card Fail*. 2019;**25**(2):87-96.
13. Shintani Y, Takahama H, Hamatani Y, Nishimura K, Kanzaki H, Kusano K, Noguchi T, Toyoda K, Yasuda S, Izumi C. Ischemic stroke risk during post-discharge phases of heart failure: association of left ventricular concentric geometry. *Heart Vessels*. 2020;**35**(4):564–575.
14. Wu CK, Lee JK, Hsu JC, Su MM, Wu YF, Lin TT, Lan CW, Hwang JJ, Lin LY. Myocardial adipose deposition and the development of heart failure with preserved ejection fraction. *Eur J Heart Fail*. *2020;****22****(3):445–454.*
15. Telles F, Nanayakkara S, Evans S, Patel HC, Mariani JA, Vizi D, William J, Marwick TH, Kaye DM. Impaired left atrial strain predicts abnormal exercise haemodynamics in heart failure with preserved ejection fraction. *Eur J Heart Fail*. 2019;**21**(4):495-505.
16. Sobirin MA., Herry Y., Sofia SN., Uddin I., Rifqi S., Tsutsui H. Effects of coenzyme Q10 supplementation on diastolic function in patients with heart failure with preserved ejection fraction. Drug Discov Ther 2019;**13**(1):38-46.
17. Lundberg A, Johnson J, Hage C, Bäck M, Merkely B, Venkateshvaran A, Lund LH, Nagy AI, Manouras A. Left atrial strain improves estimation of filling pressures in heart failure: a simultaneous echocardiographic and invasive haemodynamic study. *Clinical Research in Cardiology*. 2018;**108**(6):703-715.
18. Al Saikhan L, Hughes AD, Chung WS, Alsharqi M, Nihoyannopoulos P. Left atrial function in heart failure with mid-range ejection fraction differs from that of heart failure with preserved ejection fraction: a 2D speckle-tracking echocardiographic study. *Eur Heart J Cardiovasc Imaging*. 2019;**20**(3):279-290.
19. Burns JA, Sanchez C, Beussink L, Daruwalla V, Freed BH, Selvaraj S, Shah SJ. Lack of Association Between Anemia and Intrinsic Left Ventricular Diastolic Function or Cardiac Mechanics in Heart Failure With Preserved Ejection Fraction. *Am J Cardiol*. 2018;**122**(8):1359-1365.
20. Obokata M, Reddy YNV, Melenovsky V, Pislaru S, Borlaug BA. Deterioration in right ventricular structure and function over time in patients with heart failure and preserved ejection fraction. *Eur Heart J*. 2019;**40**(8):689-697.
21. Nagy AI, Hage C, Merkely B, Donal E, Daubert JC, Linde C, Lund LH, Manouras A. Left atrial rather than left ventricular impaired mechanics are associated with the pro-fibrotic ST2 marker and outcomes in heart failure with preserved ejection. *J Intern Med*. 2018;**283**(4):380-391.
22. Carluccio E, Biagioli P, Mengoni A, Francesca Cerasa M, Lauciello R, Zuchi C, Bardelli G, Alunni G, Coiro S, Gronda EG, Ambrosio G. Left Atrial Reservoir Function and Outcome in Heart Failure With Reduced Ejection Fraction. *Circulation: Cardiovascular Imaging*. 2018;**11**(11).
23. Malagoli A, Rossi L, Bursi F, Zanni A, Sticozzi C, Piepoli MF, Villani GQ. Left Atrial Function Predicts Cardiovascular Events in Patients With Chronic Heart Failure With Reduced Ejection Fraction. *J Am Soc Echocardiogr*. 2019;**32**(2):248-256.
24. Eroglu E, Kilicgedik A, Kahveci G, Bakal RB, Kirma C. Red cell distribution width and its relationship with global longitudinal strain in patients with heart failure with reduced ejection fraction: a study using two-dimensional speckle tracking echocardiography. *Kardiol Pol*. 2018;**76**(3):580-585.
25. Almeida P, Rodrigues J, Lourenco P, Maciel MJ, Bettencourt P. Left atrial volume index is critical for the diagnosis of heart failure with preserved ejection fraction. *J Cardiovasc Med (Hagerstown)*. 2018;**19**(6):304-309.
26. Liu S, Guan Z, Zheng X, Meng P, Wang Y, Li Y, Zhang Y, Yang J, Jia D, Ma C. Impaired left atrial systolic function and inter-atrial dyssynchrony may contribute to symptoms of heart failure with preserved left ventricular ejection fraction: A comprehensive assessment by echocardiography. *Int J Cardiol*. 2018;**257**:177-181.
27. Shah SJ., Lam CSP., Svedlund S., et al. Prevalence and correlates of coronary microvascular dysfunction in heart failure with preserved ejection fraction: PROMIS-HFpEF. Eur Heart J 2018;**39**(37):3439-3450
28. Xu B., Kawata T., Daimon M., et al. Prognostic value of a simple echocardiographic parameter, the right ventricular systolic to diastolic duration ratio, in patients with advanced heart failure with non-ischemic dilated cardiomyopathy. Int Heart J 2018;**59**(5):968-975.
29. Saha SK., Luo XX., Gopal AS., et al. Incremental prognostic value of multichamber deformation imaging and renal function status to predict adverse outcome in heart failure with reduced ejection fraction. Echocardiography 2018;**35**(4):450-458.
30. Abohammar S., ElSaidy MA., Fathalla D., Aldosarri M. Baseline characteristics of patients with heart failure and preserved ejection fraction at admission with acute heart failure in Saudi Arabia. *Egypt Heart J*. 2017;**69**(1):21-28.
31. Modin D., Sengeløv M., Jørgensen PG., et al. Global longitudinal strain corrected by RR interval is a superior predictor of all-cause mortality in patients with systolic heart failure and atrial fibrillation. ESC Hear Fail 2018;**5**(2):311-318.
32. Batalli A, Ibrahimi P, Bytyçi I, Ahmeti A, Haliti E, Elezi S, Henein MY, Bajraktari G. Different determinants of exercise capacity in HFpEF compared to HFrEF. *Cardiovascular Ultrasound*. 2017;**15**(1):12.
33. Sugimoto T, Bandera F, Generati G, Alfonzetti E, Bussadori C, Guazzi M. Left Atrial Function Dynamics During Exercise in Heart Failure: Pathophysiological Implications on the Right Heart and Exercise Ventilation Inefficiency. *JACC Cardiovasc Imaging*. 2017;**10**(10 Pt B):1253-1264.
34. Hage C., Michaëlsson E., Linde C., et al. Inflammatory Biomarkers Predict Heart Failure Severity and Prognosis in Patients with Heart Failure with Preserved Ejection Fraction: A Holistic Proteomic Approach. Circ Cardiovasc Genet 2017;**10**(1).
35. Sargento L., Vicente Simões A., Longo S., Lousada N., Palma Dos Reis R. Left atrial function index predicts long-term survival in stable outpatients with systolic heart failure. Eur Heart J Cardiovasc Imaging 2017;**18**(2):119-127.
36. Aung SM, Guler A, Guler Y, Huraibat A, Karabay CY, Akdemir I. Left atrial strain in heart failure with preserved ejection fraction. *Herz*. 2017;**42**(2):194-199.
37. Hung CL, Yun CH, Lai YH, Sung KT, Bezerra HG, Kuo JY, Hou CJ, Chao TF, Bulwer BE, Yeh HI, Shih SC, Lin SJ, Cury RC. An observational study of the association among interatrial adiposity by computed tomography measure, insulin resistance, and left atrial electromechanical disturbances in heart failure. *Medicine (Baltimore)*. 2016;**95**(24):e3912.
38. Freed BH, Daruwalla V, Cheng JY, Aguilar FG, Beussink L, Choi A, Klein DA, Dixon D, Baldridge A, Rasmussen-Torvik LJ, Maganti K, Shah SJ. Prognostic Utility and Clinical Significance of Cardiac Mechanics in Heart Failure With Preserved Ejection Fraction: Importance of Left Atrial Strain. *Circ Cardiovasc Imaging*. 2016;**9**(3): e003754.
39. Unger ED., Dubin RF., Deo R., et al. Association of chronic kidney disease with abnormal cardiac mechanics and adverse outcomes in patients with heart failure and preserved ejection fraction. Eur J Heart Fail 2016;**18**(1):103-12.
40. Georgievska-Ismail L., Zafirovska P., Hristovski Z. Evaluation of the role of left atrial strain using two-dimensional speckle tracking echocardiography in patients with diabetes mellitus and heart failure with preserved left ventricular ejection fraction. Diabetes Vasc Dis Res 2016;**13**(6):384-394.
41. Melenovsky V, Hwang SJ, Redfield MM, Zakeri R, Lin G, Borlaug BA. Left atrial remodeling and function in advanced heart failure with preserved or reduced ejection fraction. *Circ Heart Fail*. 2015;**8**(2):295-303.
42. Garcia EL, Menezes MG, Stefani Cde M, Danzmann LC, Torres MA. Ergospirometry and echocardiography in early stage of heart failure with preserved ejection fraction and in healthy individuals. *Arq Bras Cardiol*. 2015;**105**(3):248-255.
43. Hasselberg NE, Haugaa KH, Sarvari SI, Gullestad L, Andreassen AK, Smiseth OA, Edvardsen T. Left ventricular global longitudinal strain is associated with exercise capacity in failing hearts with preserved and reduced ejection fraction. *Eur Heart J Cardiovasc Imaging*. 2015;**16**(2):217-224.
44. Sanchis L, Gabrielli L, Andrea R, Falces C, Duchateau N, Perez-Villa F, Bijnens B, Sitges M. Left atrial dysfunction relates to symptom onset in patients with heart failure and preserved left ventricular ejection fraction. *Eur Heart J Cardiovasc Imaging*. 2015;**16**(1):62-67.
45. Donal E, Lund LH, Oger E, Hage C, Persson H, Reynaud A, Ennezat PV, Bauer F, Sportouch-Dukhan C, Drouet E, Daubert JC, Linde C, KaRen I. Baseline characteristics of patients with heart failure and preserved ejection fraction included in the Karolinska Rennes (KaRen) study. *Arch Cardiovasc Dis*. 2014;**107**(2):112-121.
46. Burke MA, Katz DH, Beussink L, Selvaraj S, Gupta DK, Fox J, Chakrabarti S, Sauer AJ, Rich JD, Freed BH, Shah SJ. Prognostic importance of pathophysiologic markers in patients with heart failure and preserved ejection fraction. *Circ Heart Fail*. 2014 Mar 1;**7**(2):288-299.
47. Motoki H, Borowski AG, Shrestha K, Troughton RW, Martin MG, Tang WH, Klein AL. Impact of left ventricular diastolic function on left atrial mechanics in systolic heart failure. *Am J Cardiol*. 2013;**112**(6):821-826.
48. Obokata M, Negishi K, Kurosawa K, Arima H, Tateno R, Ui G, Tange S, Arai M, Kurabayashi M. Incremental diagnostic value of la strain with leg lifts in heart failure with preserved ejection fraction. *JACC Cardiovasc Imaging*. 2013;**6**(7):749-758.
49. Carluccio E., Dini FL., Biagioli P., et al. The “Echo Heart Failure Score”: An echocardiographic risk prediction score of mortality in systolic heart failure. Eur J Heart Fail 2013;**15**(8):868-76.
50. Tan YT, Wenzelburger F, Lee E, Nightingale P, Heatlie G, Leyva F, Sanderson JE. Reduced left atrial function on exercise in patients with heart failure and normal ejection fraction. *Heart*. 2010;**96**(13):1017-1023.
51. Jaubert MP, Armero S, Bonello L, Nicoud A, Sbragia P, Paganelli F, Arques S. Predictors of B-type natriuretic peptide and left atrial volume index in patients with preserved left ventricular systolic function: an echocardiographic-catheterization study. *Arch Cardiovasc Dis*. 2010;**103**(1):3-9.
52. Hinderliter AL, Blumenthal JA, O'Conner C, Adams KF, Dupree CS, Waugh RA, Bensimhon D, Christenson RH, Sherwood A. Independent prognostic value of echocardiography and N-terminal pro-B-type natriuretic peptide in patients with heart failure. *Am Heart J*. 2008;**156**(6):1191-1195.
53. Donal E, Raud-Raynier P, De Place C, Gervais R, Rosier A, Roulaud M, Ingels A, Carre F, Daubert JC, Denjean A. Resting echocardiographic assessments of left atrial function and filling pressure interest in the understanding of exercise capacity in patients with chronic congestive heart failure. *J Am Soc Echocardiogr*. 2008;**21**(6):703-710.
54. Shah AM, Cikes M, Prasad N, Li G, Getchevski S, Claggett B, Rizkala A, Lukashevich I, O'Meara E, Ryan JJ, Shah SJ, Mullens W, Zile MR, Lam CSP, McMurray JJV, Solomon SD, Investigators P-H. Echocardiographic Features of Patients With Heart Failure and Preserved Left Ventricular Ejection Fraction. *J Am Coll Cardiol*. 2019;**74**(23):2858-2873.
55. Shah AM, Shah SJ, Anand IS, Sweitzer NK, O'Meara E, Heitner JF, Sopko G, Li G, Assmann SF, McKinlay SM, Pitt B, Pfeffer MA, Solomon SD, Investigators T. Cardiac structure and function in heart failure with preserved ejection fraction: baseline findings from the echocardiographic study of the Treatment of Preserved Cardiac Function Heart Failure with an Aldosterone Antagonist trial. *Circ Heart Fail*. 2014;**7**(1):104-115.
56. Santos AB, Kraigher-Krainer E, Gupta DK, Claggett B, Zile MR, Pieske B, Voors AA, Lefkowitz M, Bransford T, Shi V, Packer M, McMurray JJ, Shah AM, Solomon SD, Investigators P. Impaired left atrial function in heart failure with preserved ejection fraction. *Eur J Heart Fail*. 2014;**16**(10):1096-1103.
57. Oh JK, Pellikka PA, Panza JA, Biernat J, Attisano T, Manahan BG, Wiste HJ, Lin G, Lee K, Miller FA, Jr., Stevens S, Sopko G, She L, Velazquez EJ, Investigators ST. Core lab analysis of baseline echocardiographic studies in the STICH trial and recommendation for use of echocardiography in future clinical trials. *J Am Soc Echocardiogr*. 2012;**25**(3):327-336.
58. Zile MR, Gottdiener JS, Hetzel SJ, McMurray JJ, Komajda M, McKelvie R, Baicu CF, Massie BM, Carson PE, Investigators IP. Prevalence and significance of alterations in cardiac structure and function in patients with heart failure and a preserved ejection fraction. *Circulation*. 2011;**124**(23):2491-2501.
59. Gupta DK, Shah AM, Castagno D, Takeuchi M, Loehr LR, Fox ER, Butler KR, Mosley TH, Kitzman DW, Solomon SD. Heart failure with preserved ejection fraction in African Americans: The ARIC (Atherosclerosis Risk In Communities) study. *JACC Heart Fail*. 2013;**1**(2):156-163.
60. Jasic-Szpak E, Marwick TH, Donal E, Przewlocka-Kosmala M, Huynh Q, Gozdzik A, Woznicka AK, Jankowska EA, Ponikowski P, Kosmala W. Prediction of AF in Heart Failure With Preserved Ejection Fraction: Incremental Value of Left Atrial Strain. *JACC Cardiovasc Imaging.* 2021;**14**(1):131-144.
61. Carluccio Erberto EE. Fibrosis assessment by integrated backscatter and its relationship with longitudinal deformation and diastolic function in heart failure with preserved ejection fraction. *International Journal of Cardiovascular Imaging*. 2016;**32**(7):1071-1080.
